# Asbestos as a Surgical Dressing

**Published:** 1896-09

**Authors:** 


### Asbestos as a Surgical Dressing.

Dr. E. O'N. Kane, of Kane, Pa., recommends asbestos as a practical and useful substance for surgical dressings. These dressings, he says, may be carried in any parcel, paper bag, or hand-satchel, may be handled by dirty hands, spattered by blood or any sort of filth, and yet can be rendered absolutely aseptic in less than two minutes by tossing them upon the coals or into the blaze of an ordinary kitchen stove. After having completed an operation, and just before he is ready to apply the dressings, they are thrust into the coals or flame of the nearest stove. The same dressings can be used, if necessary, though here it is advisable to wash off some of the discharges before the dressings are burned. Repeated burnings seem to injure the quality of the material somewhat. The form of asbestos most used is the asbestos fibre, which is as soft as silk floss, and its absorbent qualities are greater than those of absorbent cotton. Asbestos wicking, packing, and cording are adapted for drainage tubes.—*Medical Record.*

